# *Anopheles* control is considerably more complicated than *Aedes* control

**DOI:** 10.1590/0037-8682-0428-2019

**Published:** 2020-01-27

**Authors:** Djane Clarys Baia-da-Silva, Gisely Cardoso de Melo, Paulo Pimenta, Marcus Vinícius Guimarães de Lacerda, Wuelton Marcelo Monteiro

**Affiliations:** 1 Fundação de Medicina Tropical Dr. Heitor Vieira Dourado, Manaus, AM, Brasil.; 2 Universidade do Estado do Amazonas, Programa de Pós-Graduação em Medicina Tropical, Manaus, AM, Brasil.; 3 Fundação Oswaldo Cruz, Instituto de Pesquisas René Rachou, Belo Horizonte, MG, Brasil.; 4 Fundação Oswaldo Cruz, Instituto de Pesquisas Leônidas and Maria Deane, Manaus, AM, Brasil.

Dear Editor:

The letter by Dr. Wermelinger^1^ doi: 10.1590/0037-8682-0385-2019 provides an interesting critique of the evolution of vector-borne disease control in Brazil, highlighting the need to invest in new environment-friendly control methods that are economically viable and feasible to implement. 

In terms of innovative methods for vector control involving interaction between mosquitoes and bacteria, greater advances have been made in controlling dengue than malaria due to the differing biology of the respective vectors.

Previous studies on the anopheline microbiome indicate that there is no natural obligate endosymbiont in the *Anopheles* genus, which is in contrast to what has been observed in some *Aedes* species that are susceptible to *Wolbachia*. This susceptibility, along with the fact that colonized mosquitoes are refractory to dengue virus infection, represents an exciting potential new form of biocontrol for arboviral diseases, including dengue. Strains of *Wolbachia*, deliberately introduced into *Ae. aegypti* mosquitoes, have been shown a capacity to spread in high frequencies within mosquito populations in release trials, and mosquitoes infected with these strains have shown markedly reduced vector competence^2^ ^,^ ^3^ ^,^ ^4^. In *Anopheles*, it is possible that secondary symbionts may have become recently more susceptible to anophelines and are, therefore, not obligate^5^, although they may fulfill a role in host biology and susceptibility to *Plasmodium* ^*6*^.

Wermelinger’s letter also highlights the need to consider methodological principles that should guide control interventions, with an emphasis on integrated vector management. This appears possible using *Wolbachia* in *Ae. aegypti* since *Wolbachia* also blocks other circulating arboviruses such as Chikungunya and Zika viruses^6^ ^,^ ^7^. Regarding *Anopheles*, only *Plasmodium* parasites are transmitted, making it difficult to plan joint control strategies involving other diseases.

*Ae. aegypti* is an extremely anthropophilic mosquito, frequently found in urban areas, that lives in or around households or other buildings frequented by people, such as businesses and schools. *An. darlingi*, the most important vector of malaria in Brazil, is the most frequent anopheline found within human housing. This mosquito is particularly aggressive towards humans, usually attacking people inside their homes in the early evening. *Ae. aegypti* mosquitoes prefer to *breed* in *areas* such as stagnant water and in artificial containers such as flower vases, uncovered barrels, buckets, and discarded tires, whereas *An. darlingi* use natural and artificial water bodies, such as ponds, riverbanks, puddles, and water-logged fields, preferably involving clean water comprising organic matter, aquatic vegetation, and shade. Therefore, anophelines are much more exposed to other environmental bacteria that could compete with *Wolbachia*. In addition, malaria also occurs in rural, riverine, and indigenous areas, where the maintenance of engineered vector populations may only last for short periods of time. 

All these factors are likely to help explain studies indicating the usefulness of artificially manipulated microbiome mosquitoes in relation to dengue fever and the absence of similar field studies concerning malaria. Recently, the Brazilian Ministry of Health, in partnership with Fiocruz’s World Mosquito Program-Brazil, as part of a campaign against dengue, Zika, Chikungunya, and yellow fever, announced the expansion of the *Wolbachia* method into three Brazilian cities, Campo Grande (MS), Belo Horizonte (MG), and Petrolina (PE)^8^. This is a field where malaria researchers are only in the early stages of basic research; further studies are needed to achieve a breakthrough.

Currently, promising entomopathogens remain restricted to *Bacillus thuringiensis* and *B. sphaericus*. Even for the available strains, information concerning their action in the field is scarce in the Amazonia^9^, but it indicates that these biolarvicides may be effective for larval source management aiming at malaria control^9^ ^,^ ^10^. Furthermore, dengue has overtaken malaria in terms of the volume of research concerning this issue. A PubMed search (accessed on August 28, 2019) using the terms *Bacillus thuringiensis* OR *Bacillus sphaericus* and *Aedes* OR dengue retrieved 676 publications, whereas using the terms *Bacillus thuringiensis* OR *Bacillus sphaericus* and *Anopheles* OR malaria identified 384 publications.

To reduce malaria transmission, a successful programmer needs to focus on several aspects, including early diagnosis, use of effective antimalarial drugs, and vector control^11^. These strategies are critical for malaria elimination. In this context, tools such as genetically modified mosquitos, transmission-blocking strategies (using drugs, antibodies, or even *Wolbachia*-like microorganisms), and breeding site management with bioinsecticides should be considered. However, apart from the unpublished experiences of individual malaria control programmers and unlike the situation with dengue, there is a lack of robust evidence supporting the efficacy of specific tools for malaria control and elimination through employing vector control measures.
